# The Stable

**Published:** 1866-04

**Authors:** 


### The Stable

Should be arranged to be above ground, to be well ventilated, and to have abundant light; in short, to be cool in summer and warm in winter. He can never be a successful farmer who does not shelter his cattle effectually and well, in all seasons from the inclemencies of the weather. It is not only a humanity, but a great pecuniary saving on every farm where there is a single living animal. Some build stables low for warmth, but the advantage is more than lost by the vitiation of the atmosphere. A warm, bad air is worse than the cooler and still atmosphere of a stable. The ceiling of a stable should be at least ten feet high, with an aperture for the escape of foul air; the walls or partitions should be close, and arranged to have abundant light admitted through glass windows. In summer the sash may be removed.

The *American Agriculturist* for December, 1863, gives a description of a stable for draught and farm horses which contains the most important points on this subject:

"The stable should not be less than eighteen feet wide, and of such a length as will allow six feet standing for each horse. It should be ten feet high. The horses stand in a single row, and the harness is hung on pegs in the wall behind them. This width admits of thorough ventilation to the stable, without subjecting the horses to drafts. Each standing should be parted off by an upright post reaching from the ground to the ceiling rafter, placed three feet from the wall at the horse's head. These partitions should be closely boarded up three feet above the manger and hay-crib, to prevent the horses quarreling about the food, and biting each other. To each of these posts a 'bale,' eight feet long and twenty inches wide, should be hung by a strong chain, to divide the standings, and suspended by another strong chain at the hinder end from the ceiling rafter. Each chain should have a hook and eye within reach, that may be readily unfastened. This arrangement will leave a space of six feet opposite the head of each horse, available for feeding purposes. The manger for corn and chaff (cut feed)

may be two and a half feet long. It should be two feet wide at the top, one foot two inches at the bottom. The hay and straw, which should be cut into six-inch lengths, will require a larger receptacle, which should be three feet six inches long, two feet wide at its upper part, and half that width below. It should be so constructed, that while it is even with the manger above, it should reach to the ground, two feet above which should be fixed to the wall a bottom, sloping to one foot above the ground in the front, where some upright openings should be cut, to allow the escape of the seeds and dirt.

"At the top of this hay and straw-crib, an iron rack with bars six inches apart, should be so hung as to open up and fall back against the wall to let the fodder be put in, and then be put down upon it for the horse to eat through. It should be so much smaller than the opening that it can fall down with the fodder as it is consumed, by which means not a particle is wasted. The manger may be constructed of yellow deal one and a half inches thick for the front, back, and ends; the bottom, of slate three quarters of an inch thick. The top of the front and ends should be covered with half round iron, two and a half inches wide, screwed on to project over the front, a quarter of an inch outside, and three quarters of an inch inside the manger. This prevents the food being tossed out and the manger being gnawed. A short post must be put up as near the center of the standing as possible, to support the manger, into which a large screw ring must be put to let the chain or rope of the headstall pass freely up and down without constant friction. The manger may be three and a half feet from ground to top; the hay-crib of course the same hight. The paving of the standings, to three and a half feet from the head, should be flat, then with a fall from both sides to the center, where an angle iron drain of four inches wide from end to end, with a removable flat iron cover fitted to the inside of it, should be placed straight down the standing, with a fall into another larger cross main drain ten feet six inches from the head, so placed as to carry away the urine from all the smaller drains into a tank outside the stable. This main drain so placed, takes the urine from the mares, and has a loose cover also fitted to it, easily removed for sweeping out when necessary, perhaps once a week. This system keeps the stable healthy, economizes the urine, and the straw also—the latter very important where it can be sold, or consumed as food. The width of eighteen feet for the stable gives room for narrow corn bins three feet high, so that each carter may have his horses' corn separate."

In the above, paving has been alluded to for standings, but a hard, dry dirt floor is greatly better than stone or plank. A nice smooth, hard, and dry floor may be secured with small stones packed like a McAdamized road, the interstices being filled up with good cement, or with the dust made by breaking up limestone rock. This will make a floor which water can not penetrate nor horse-shoe disturb. The cheapest and best bedding, at least near mills, for such a floor, or for any other if kept dry, is saw-dust, which should be laid in abundantly, when dry, in the fall of the year.

It may be added that a good farmer and a generous man, having arranged his house for the comfort, health, and happiness of his family and the elevation of the tastes of his neighborhood, will not rest satisfied as long as the noble horse, the useful cow, and the patient ox and mule are without comfortable quarters, warm in winter, cool in summer, and all the year round abundantly fed and kindly treated, extending these with a right good-will to pigs and poultry too!

